# Roundtable on Young Adults with IBD: A multi-stakeholder perspective and patient-driven analysis of the current transitional challenges and gaps in research

**DOI:** 10.1016/j.hctj.2023.100006

**Published:** 2023-06-28

**Authors:** Sneha Dave, Sydney Reed, Kajal Patel, Sandra C. Kim

**Affiliations:** a Generation Patient, USA; b Herbert Wertheim College of Medicine, USA; c Department of Pediatric Gastroenterology, Hepatology and Nutrition, Cleveland Clinic Children’s, USA

## Abstract

The “Roundtable on Young Adults with IBD” is a year-long learning community consisting of monthly discussions between providers, patients, and healthcare partners. The group aims to enhance outcomes for the young adult IBD patient population. The first discussion centered on the difficulties that IBD patients face during the transition from pediatric to adult care and how care can be improved for young adult patients during this phase. The group discussed the need to identify developmental milestones for young adult patients, further screening for disordered eating, and the importance of collaboration between specialists and pediatric and adult providers for a successful transition. Additionally, the group highlighted areas of concern such as the spread of misinformation through social media, social determinants of health, and the potential lifelong psychosocial, mental, and economic burdens of IBD for the young adult patient population.

## Introduction

The Roundtable on Young Adults with IBD is a year-long learning community comprised of monthly discussions between patients and providers which seek to improve outcomes for the young adult (YA) IBD patient population. Each monthly discussion will focus on a pressing issue among the YA IBD demographic. Our first Roundtable session began with an initial introductory presentation facilitated by Sneha Dave, a YA IBD patient, and Sandra Kim, the Chair of Pediatric Gastroenterology, Hepatology, and Nutrition at the Cleveland Clinic. This presentation focused on understanding emerging adults with IBD and was followed by a discussion between five YA patients with IBD, four care partners, and five medical professionals. A central theme throughout this discussion involved the challenges for patients living with IBD as they transition from pediatric to adult care, specifically regarding how care can be changed for adolescents and young adults undergoing this transition within the hospital/office setting and beyond. This was a critical point of reflection throughout the conversation.

The transition process from pediatric care to subsequent transfer to adult care, though it can vary greatly among individual patient circumstances (geographical, socioeconomic, etc.), is crucial in enabling patients to succeed beyond childhood. It is essential to understand that this difficult process usually coincides with other major life transitions and can span several years, depending on the patient. Failure to effectively transfer care can result in significant consequences, such as delays and gaps in treatment that may significantly impact the patient's health and resilience, other areas of transition, and the patient’s overall life trajectory. Data supporting this assertion describes failures of transition services as patients transfer care to the adult-centered medical system, including high rates of emergency care utilization amongst adults ages 20–29 years and increased pediatric hospitalizations for young adults with chronic conditions.^1^

## Importance of “receivership” and collaboration in the transition of care for young adults with IBD

Pediatricians and subspecialists are typically well-versed in transition, but this is often less true of the adult physicians to whom YA patients are being transferred. These doctors are often less informed or focused on this age demographic's issues. When speaking of her transition, one YA patient described her struggle to find physicians prepared to take on a patient with complex care needs and her reliance on a creative primary care physician (PCP) to help her navigate comorbid medical conditions. Several YA IBD patients expressed a need for general pediatricians and pediatric gastroenterologists to work together to facilitate the transitioning process for their patients as they identified appropriate primary and specialty care providers in adult medicine. This sentiment is supported by research in which caregivers and adolescents indicated that ideal comprehensive care would involve a partnership between primary care and GI providers.^2^ One physician stressed the importance of “receivership,” which she defined as connecting with adult-care colleagues who are passionate and knowledgeable about YA patients and prepared to receive them and help complex patients through this transition-transfer process.

While some YA patients may have the option to stay with their pediatric physicians beyond childhood, one pediatric gastroenterologist noted there are several aspects of adult care that pediatric physicians have less experience in their practice, underscoring why it is important for young adult patients to be seen by adult physicians. Examples include colon cancer screenings, fertility challenges and concerns, and familiarity with medications only approved for adults with whom pediatric specialists may have less experience and access. Therefore, it is important to establish an effective overall transitioning process while a patient is still in pediatric care that is purposeful and planned, emphasizing a structured transition program rather than simply transferring care from one healthcare provider to another, to ensure successful care transfer.^3^

## A need for more formal support measures during the transition to adult care

One parent of a YA with IBD recounted her experience as a caregiver to her child during this transition period. She expressed feeling the expectation of genuine success was unrealistic for patients and families managing multiple care transfers without adequate support. She detailed what patients are expected to navigate by themselves, including insurance, managing appointments, changing healthcare professionals, and ordering specialty medications. This was compounded by stressors including starting college and/or a new job and independent living, especially if the YA patient’s disease was under inadequate control. Furthermore, as noted by the parent, resources (even at a well-established IBD program at a major medical center), were limited for patients and families transitioning and transferring to adult care. While a medical social worker was available to offer some assistance, no formal transition coordinator could adequately help them navigate this process. As defined by experts in adolescent medicine, effective transitioning for adolescents with IBD requires “adequate resources and/or a specialized transition program designed to facilitate and support the process”.^4^ Furthermore, “transition programs or designated transition clinics are unique and offered at only a handful of centers across the United States and abroad,” a fact that is still true nearly nine years later.^4^

## Gaps in research regarding milestones for the young adult, measuring resilience, and defining success

The difficulty physicians currently face in understanding how young adult patients are truly coping was acknowledged by one practitioner. Further gaps in knowledge were evident as little research has been done with regard to the developmental milestones of the young adult. Another physician concluded by explaining that physicians don’t know what outcomes to measure and how those outcomes might predict who is or is not going to do well after they transfer care. This doctor asked, “As physicians when we say that someone has successfully transferred to adult care, how do we define success?” There was group consensus that more research should be done around measuring resilience for this age demographic along with a need to create more detailed milestone criteria for adolescents and young adult patients. Numerous published checklists for milestones and/or accomplishments for pediatric patients exist, but most end at ages 18–20. It was noted that a more ideal age to end such a checklist would be 25 years to ensure future success and quality of life beyond pediatric specialty care. It should also be noted that some studies focusing on YA with IBD utilize the broader definition of emerging adulthood (ages 18–29 years) given the impact of ongoing IBD management on prolonging the transition period.^5^

## Increased awareness and screening for disordered eating in young adults with IBD

Numerous YA patients disclosed issues surrounding disordered eating resulting from living with IBD. One patient expressed her belief that most young-adult patients living with IBD likely have dealt with some form of disordered eating due to their condition and treatment. Patients described the combination of defined and/or restrictive diets and weight fluctuations with the loss of control over what one can and cannot eat as a contributing factor in disordered eating patterns. This is compounded by the fact that many social interactions during young adulthood center around food, making this issue even more complicated during this period of life. One physician affirmed that there is substantial research to confirm that the rates of body image concerns and disordered eating are higher among YA patients with IBD compared to the general population,^6^ and that these issues are common in other diseases in which food is involved.^7^ Furthermore, living with IBD can contribute to body image issues by affecting the way a person’s body looks as a result of surgeries and medication side effects (i.e. corticosteroids). YA patients felt that it was extremely important for IBD psychologists, physicians, and nurses to be aware of this information by using language that does not reinforce negative impacts or behaviors. While awareness is the first step, patients and physicians expressed a need for more appropriate interventions for this issue and universal screening measures.

## Social determinants of health

As defined by the CDC, the social determinants of health are the nonmedical categories that affect health outcomes^8^. The five categories commonly recognized are healthcare access and quality, education access and quality, social and community context, economic stability, and neighborhood and built environment. The three social determinants in our roundtable discussion were health outcomes, equity of treatment, and fostering care. Our discussion highlighted the belief that YA patients with IBD may not feel that their care transition process to prepare for the transfer of care from pediatric to adult GI providers was adequate, potentially negatively influencing health outcomes. A meta-analysis on the association between trust in healthcare professionals and health outcomes highlighted that patients reported greater health behaviors, less relapse, and a better quality of life when developing a trustworthy doctor-patient relationship.^9^ If this key link is missing in the care transition, the patient's overall well-being may be at risk. Additionally, equity of treatment must not be mistaken for equality of treatment, which is defined as the quality of being fair and realizing that each patient has different circumstances.^10^ For instance, a well-supported patient with access to an IBD center may not have the same health outcomes as a patient who lives in poverty and of lower socioeconomic status and therefore, may not have access to the same resources. These differences in health equity need to be addressed and may involve targeting certain demographics through online resources or targeted seminars and conferences to reach patient populations that may otherwise have been overlooked. Fostering care, as discussed throughout the roundtable, involves a medical team working together. A patient in our discussion highlighted how managing their care between providers created an extra burden. For example, they not only had to navigate their own illness but coordinate care between their physicians when it came to disease management and medication reconciliation. Healthcare providers from different specialties need to work together to cultivate a healthy environment for patients.

## Misinformation and disinformation epidemic

In today’s day and age, social media consumes a substantial amount of time for the average consumer, especially young adults. On one hand, social media offers IBD patients the opportunity to connect with others; however, it can also have a detrimental effect on patients. Based on a study completed in 2019, most patients with IBD used Facebook as their main reference source for information about IBD. From the cross-sectional study, fifty percent of patients were not sure of the quality of IBD information that was presented online^11^ and concluded the importance of decreasing misinformation by having healthcare providers guide their patients on where and how to access reliable resources online. In conjunction with transitioning care, members of the Roundtable suggested that adult gastroenterologists need to be aware of how social media may impact young adults compared to the general adult population.

## Further Considerations for the Global Context of Young Adults with IBD

YA patients living with IBD face many lifelong psychosocial, mental, and economic burdens, including such psychosocial issues as isolation and stigma. Compared to the general population, patients with IBD are 3–5 times more likely to develop anxiety disorders and two to four times more likely to experience depression.^12^ Financially, patients face the cost of surgeries, medications, and hospitalizations, which insurance may not fully approve. Young adults face a unique challenge as most of them are dealing with a chronic illness during the beginning of their careers, putting them on an unequal playing field compared to their peers. Navigating career paths or higher education with a chronic illness can lead to periods of absenteeism from work or education and lost productivity, adding to the patient’s mental burden. Young adults with these conditions grow up differently from their peers and face challenges that most of the general population would not consider. Additionally, YA patients with chronic illnesses face different burdens and grow up differently than young-adult patients with a curable condition. For example, a patient with an incurable condition such as IBD may need to experiment with medications before they reach remission or may in fact never reach remission because the treatments for IBD are not cures. Compare this to a child who is diagnosed with urinary tract infection for example, their treatment plan with antibiotics will lead to a complete resolution of symptoms within a given period of time. The same cannot be said for those living with incurable illnesses, as they are constantly facing the burden of potential relapse.

## Conclusion

The Roundtable on Young Adults with IBD is a year-long program that seeks to improve the quality of life and outcomes for patients living with IBD. Our first roundtable brought together patients, care partners, and healthcare providers, and the central theme highlighted the transition of care from pediatric to adult. The discussion focused on the importance of receivership, formal support measures, gaps regarding milestones, eating disorders, and additional areas of concern, including the social determinants of health and social media. The inability to professionally transfer care can lead to detrimental and long-term effects on patient outcomes. These critical issues can be addressed by developing a successful transition program designed to facilitate receivership and coordinate care to ensure a smoother, more effective transfer of care process between pediatric to adult gastroenterologists. YA patients with IBD face greater challenges compared to their peers, and addressing these issues is critical for improving the lives of this patient population.

## Human rights

This article does not contain any studies with human participants performed by any of the authors.

## Informed consent

This article does not involve human participants, so informed consent was not required.

## Welfare of animals

This article does not contain any studies with animals performed by any of the authors.

## Funding

This work was funded by 10.13039/100007028The Leona M. and Harry B. Helmsley Charitable Trust10.13039/100007028.

## Declaration of Competing Interest

Sydney Reed, Sneha Dave, Kajal Patel, and Sandra Kim declare that they have no conflicts of interest.
